# Dynamic Visualization of Dendritic Cell-Antigen Interactions in the Skin Following Transcutaneous Immunization

**DOI:** 10.1371/journal.pone.0089503

**Published:** 2014-02-24

**Authors:** Teerawan Rattanapak, James C. Birchall, Katherine Young, Atsuko Kubo, Sayumi Fujimori, Masaru Ishii, Sarah Hook

**Affiliations:** 1 School of Pharmacy, University of Otago, Dunedin, New Zealand; 2 School of Pharmacy and Pharmaceutical Sciences, Cardiff University, Cardiff, United Kingdom; 3 Laboratory of Cellular Dynamics, Immunology Frontier Research Center, Osaka University, Osaka, Japan; 4 Department of Immunology and Cell Biology, Graduate School of Medicine and Frontier Biosciences and Laboratory of Cellular Dynamics, Immunology Frontier Research Center, Osaka University, Osaka, Japan; Oklahoma Medical Research Foundation, United States of America

## Abstract

Delivery of vaccines into the skin provides many advantages over traditional parenteral vaccination and is a promising approach due to the abundance of antigen presenting cells (APC) residing in the skin including Langerhans cells (LC) and dermal dendritic cells (DDC). However, the main obstacle for transcutaneous immunization (TCI) is the effective delivery of the vaccine through the stratum corneum (SC) barrier to the APC in the deeper skin layers. This study therefore utilized microneedles (MN) and a lipid-based colloidal delivery system (cubosomes) as a synergistic approach for the delivery of vaccines to APC in the skin. The process of vaccine uptake and recruitment by specific types of skin APC was investigated in real-time over 4 hours in B6.Cg-Tg (Itgax-EYFP) 1 Mnz/J mice by two-photon microscopy. Incorporation of the vaccine into a particulate delivery system and the use of MN preferentially increased vaccine antigen uptake by a highly motile subpopulation of skin APC known as CD207^+^ DC. No uptake of antigen or any response to immunisation by LC could be detected.

## Introduction

Transcutaneous immunization (TCI) is a novel approach to deliver peptide, proteins, DNA or viral particulate vaccines into the skin to the abundant resident antigen presenting cells (APC) such as Langerhans’s cells (LC) in the epidermis and dermal dendritic cells (DDC) in the dermis. LC are distributed throughout the epidermis and make up approximately 3–5% of the total cells present [1] and have been reported to be regulators of both tolerance and immunity [2]. DDC reside in the dermis and are assumed to be key regulators of cutaneous adaptive immune responses [3]. LC have traditionally been discriminated from DDC by their location, the presence of Birbeck granules and their high langerin (CD207^+^) expression [4]. However CD207^+^ expression has also been observed on DDC populations [5]. It has been reported that these CD207^+^ DC are involved in cross-presentation of antigens and can potentially stimulate antigen-specific cytotoxic T lymphocyte (CTL) and T helper 1 cell (Th1) immune responses [6], [7], [8] whereas CD207^−^ DDC were found to induce a primarily CD4^+^ T cell response [9].

The major barrier for transcutaneous delivery of vaccines is the outer protective layer of skin; the stratum corneum (SC). One of the most common methods for enhancing vaccine permeation into skin is the utilization of lipid vesicles [10] for example, cubosomes [11]. Cubosomes are a dispersion of the cubic liquid crystalline phase that retain the unique nanostructure of the parent cubic phase; a highly twisted, continuous lipid bilayer and two congruent non-intersecting water channels [12]. It has been reported that the penetration enhancing effect of cubosomes is due to the lipids of the particles forming a mixture with the lipids of the SC due to their similar cubic phase structure and thereby fluidizing the SC [13], [14]. Cubosomes have been used successfully for the transdermal delivery of several drugs including indomethacin [14] and hydrophilic plant extracts from *Berberis koreana* [15].

Another approach to overcoming the barrier provided by the SC is to transiently perforate skin using microneedles (MN). The short needles (less than 1 mm) in microneedle arrays disrupt the SC, forming transient micro-channels that facilitate the delivery of drugs [16], macromolecules [17], [18] or vaccines [18], [19] to the deeper tissue. MN possess the potential to revolutionise the field of vaccine delivery due to low manufacturing and product distribution costs [20], the fact they may not require skilled vaccine-administration expertise, are painless to use [21] and will likely have wide public acceptance [22]. Numerous studies have investigated the ability of MN to deliver vaccines into the skin [23], [24], [25] demonstrating the effectiveness of this approach as compared to subcutaneous [26], [27] or intramuscular vaccination [24], [28]. In this study, a combination of MN and cubosomes was therefore employed to overcome the barrier imposed by the SC and enhance vaccine delivery into the skin.

While TCI has been investigated by a number of different research groups, no studies have been carried out investigating the dynamics of interactions between vaccines and APC at the cellular level in real-time and in living animals. As it has been reported that the different APC subsets in the skin may promote qualitatively different immune responses, such interactions may be crucial in determining the outcome of TCI and by understanding these interactions it may be possible to design more effective vaccines. This lack of such data is due to the limitations of traditional imaging techniques such as fluorescent microscopy and confocal laser scanning microscopy (CLSM) which have a reduced ability to visualize into deep turbid tissue [29], [30]. However, a combined approach utilizing advanced fluorescent labelling techniques and two-photon microscopy (2 PM) has been widely used in structural and functional studies of deep tissue [31]. Another key advantage of 2 PM over CLSM is reduced photo-breaching at out-of-focal plane regions [32]. Additionally, second harmonic generation (SHG) occurs concurrently with 2 PM [33]. SHG is not generated from absorption but from hyper-Rayleigh scattering by the two low energy photons [34]. SGH occurs when light interacts with asymmetric macromolecular arrangements such as collagen fibres [35]. Consequently, the overall purpose of this study was to visualize TCI in real-time with particular emphasis on interactions with different APC populations in the skin using 2 PM and also to examine the ability of a combined approach of cubosomes and MN to induce immune responses.

## Materials and Methods

### Ethics Statement

All experiments were approved by the Animal Ethics Committee, University of Otago or by the Animal Experimental Committee of Osaka University.

### Materials

Phytantriol was purchased from A & E Connock (Hampshire, England). Poloxamer 407 (Lutrol® F127) was obtained from BASF (Ludwigshafen, Germany) and 1,2-propandiol (Propylene Glycol, purity≥99.5%) from Merck (Darmstadt, Germany). CD8 Ovalbumin peptide (SIINFEKL, OVA_257–264_) and CD4 Ovalbumin peptide (ISQAVHAAHAEINEAGR, OVA_323–339_) and 5(6)-tetramethylcarboxy rhodamine-labelled OVA_257–264_ (TMR-SIINFEKL) was purchased from Mimotopes (Clayton, Australia). Monophosphoryl Lipid A from Salmonella Minnesota RE 595 was purchased from Sigma-Aldrich (Missouri, USA). Purified saponin (Quil-A) was purchased from Brenntag Biosector (Frederikssund, Denmark). All other chemicals were of analytical grade.

### Microneedle Arrays

Microneedle arrays fabricated from biocompatible polycarbonate polymers using an injection moulding process were supplied by Professor James Birchall (School of Pharmacy and Pharmaceutical Sciences, Cardiff University, UK). The 13-needles (670-µm/6 needles, 520-µm/6 needles and 335-µm/1 needle) were arranged in concentric circles [36].

### Cubosome Preparation

Antigen/adjuvant-loaded cubosomes with a size of 158 to182 nm were formulated using the lipid precursor method [37]. Briefly, phytantriol, poloxamer 407, propylene glycol and MPL were weighed into a 20-mL scintillation vial and dissolved in 5 mL chloroform. Rhodamine-labeled cubosomes were prepared by adding octadecyl rhodamine B chloride (R-18, Invitrogen Molecular Probes, OR, USA) into the scintillation vial prior to preparation of the cubosomes with a mass ratio of dye to lipid of 1∶2000. Chloroform was evaporated under a stream of nitrogen at 45°C. The concentrated actives solution (0.8 mg/20 µL Quil-A (QA) and 2 mg/20 µL peptide) and glass beads were added and mixed by shaking until visually homogenous. Milli-Q water (45°C) was gradually added into the scintillation vial to a final volume of 1 mL while vortexing for 10 min. Peptide entrapment was approximately 23% as previously reported [38].

### Mice

For 2 PM experiments, 6–10 week old male and female B6.Cg-Tg (Itgax- Enhanced Yellow Fluorescent Protein) 1 Mnz/J mice originally purchased from The Jackson Laboratory (Maine, USA) were maintained under specific pathogen-free conditions at the Osaka University animal facility under an approved protocol from the Animal Experimental Committee of Osaka University. For other studies, male C57BL/6, 6–8 weeks old were utilized, bred and maintained under specific pathogen-free condition at the HTRU, Dunedin, New Zealand.

### Two-photon Microscopy

Prior to performing the imaging experiments, the hair on the lower back of mice was removed with an electronic hair clipper. Any remaining hair was removed using depilatory cream (Nair®, Carter Products, NY). The skin was wiped with moist cotton to remove all the depilatory cream.

The imaging system was an A1RMP multiphoton microscope (Nikon, Japan) driven by a Chameleon Vision II Ti:Sapphire laser (Coherent, CA, USA) tuned to 920 nm with an inverted microscope equipped with a 20× oil immersion objective (CFI-Plan-Fluor, N.A. 0.75, Nikon). Laser power at the acquisition area was 25 mW. Fluorescent probes were detected through bandpass emission filters. Formulations were detected using a 561/25 nm filter and collagen using a 440/25 nm filter. Images were acquired from 20 to 45 z-planes, spaced 5 µm apart every 5 min for up to 240 min. Snapshot images were acquired using scanning averages of 2 scans. Raw imaging data was processed with Imaris software (Bitplane AG, Zürich, Switzerland). To assess vaccine penetration, 2 PM images were captured in the XZ-plane using the ortho slicer function in the Imaris software. The microscope system was enclosed in an environmental compartment in which anesthetised mice were warmed by heated air.

Formulations (diluted 1∶20 in sterile MilliQ-water) were applied onto the skin with excess formulation being removed after 2 min by capillary action using a tissue. For experiments utilizing MN, the MN were applied manually to the prepared skin before application of formulations. A glass slide was attached to the skin using tissue glue (Vetbond Tissue Adhesive, 3 M Animal Care Products, MN, USA). Mice were then placed on a custom-designed stage. Mice were anesthetised with isoflurane (Escain; 2% vaporized in 100% oxygen) throughout the experimental period.

### *In vivo* Uptake of Formulations by Skin LC and DC

The immunization procedures were similar to that described in the previous section. However, the testing area was protected by paper and then covered with an occlusive dressing and the cage was covered to protect from light. After euthanasia, a section of the immunization site was rinsed with PBS to remove residual formulation and then harvested. The skin was macerated and incubated at 37°C for 1 hr in collagenase type IA (C9891, Sigma-Aldrich, St. Louis, MO) digestion buffer. A single cell suspension was fixed in 8% paraformaldehyde at room temperature. Cells were then permeabilized using saponin (S-7900, Sigma-Aldrich, St. Louis, Mo) buffer and incubated with anti-CD16/CD32 before being stained with anti-CD207-Alexa Flour 488 (eBioscience, San Diego, CA) for 30 minutes followed by washing and staining with anti-CD11c-APC, anti-CD11b-PE-Cy7 (BD PharMingen, San Diego, CA) and anti-F4/80- Brilliant Violet 421 (Biolegend, San Diego, CA). Cells were then analysed using a FACSCanto™ II flow cytometer (BD Biosciences, San Jose, CA). Data was analysed using FlowJo 7.6 analysis software (Tree Star, Inc., Oregon, USA).

### Transcutaneous Immunization

Adoptive transfer was performed as described previously [39]. Briefly 4×10^6^ lymphocytes from OT-I and OT-II were injected into the tail vein of each recipient C57BL/6 mouse a day prior to TCI. The following day the C57BL/6 mice were anaesthetised and were close clipped. Self-adhering foam base (Reston™, 3 M), was attached on the skin of mouse to form a reservoir. Vaccine formulations, 100 µL containing 100 µg of CD4^+^ peptide, 100 µg of CD8^+^ peptide, 40 µg of QA and 20 µg of MPL formulated in cubosomes (2 mg of lipid) or in Milli-Q water or cubosomes containing adjuvants only (control) were applied on the skin inside the reservoir which was then covered with an occlusive dressing (Opsite-Flexifix™, Smith & Nephew Medical Limited and Blenderm™, 3 M). For experiments utilizing MN, the microneedle array was applied manually to the prepared skin before application of formulations. The reservoirs were removed after 6 hr. The immunization procedure was repeated at day 14 and then at day 18 the mice were sacrificed and regional lymph nodes taken. Single cell suspensions were then prepared from all harvested lymphoid tissues. Aliquots of single cell suspensions of lymphocytes were incubated with anti-CD16/CD32 before being stained with anti-CD4-V500, anti-CD8-PE-Cy7, anti-Vα2-PE and anti-Vβ5-biotin. This was followed by staining with SA-Percp-Cy5.5 and propidium iodide. Antibodies and propidium iodide were purchased from BD Biosciences Pharmingen (California, USA). Cells were analysed on a FACSCanto™ II (BD Biosciences) and data was analysed using FlowJo 8.6 (Tree Star Inc).

### Interferon-γ Assay

A 100 µL aliquot of a single cell suspension of 2×10^6^ lymphocytes/mL was plated in triplicate a 96-well round bottomed plate for restimulation with anti-CD3 (10 µg/mL), OVA (20 µg/mL) and IL-2 (2 ng/mL) or IL-2 alone (2 ng/mL). The plates were incubated for 3 days in a CO_2_ incubator (5% CO_2_ at 37°C) (HERAcell incubator, Heraeus, Hanau, Germany). On day 4, 100 µL of the cell culture supernatant was removed and stored at −20°C until analysis. Interferon γ was measured using a BD™ Cytometric Bead Array (CBA) Mouse T Cell Cytokine Kit according to the manufacturer’s instructions.

### Data and Statistical Analysis

Data analysis was carried out using Microsoft Excel, Version 2010. The results are expressed as mean ± SD and mean ± SEM. GraphPad Prism Version 5.0 was used to examine the statistically significant differences using the two-tailed unpaired Student’s t-test or the one-way analysis of variance (ANOVA) followed by Tukey’s pairwise comparison.

## Results

### DDC, not LC, Respond Initially to Transcutaneously Applied Antigen

**Figure 1:**
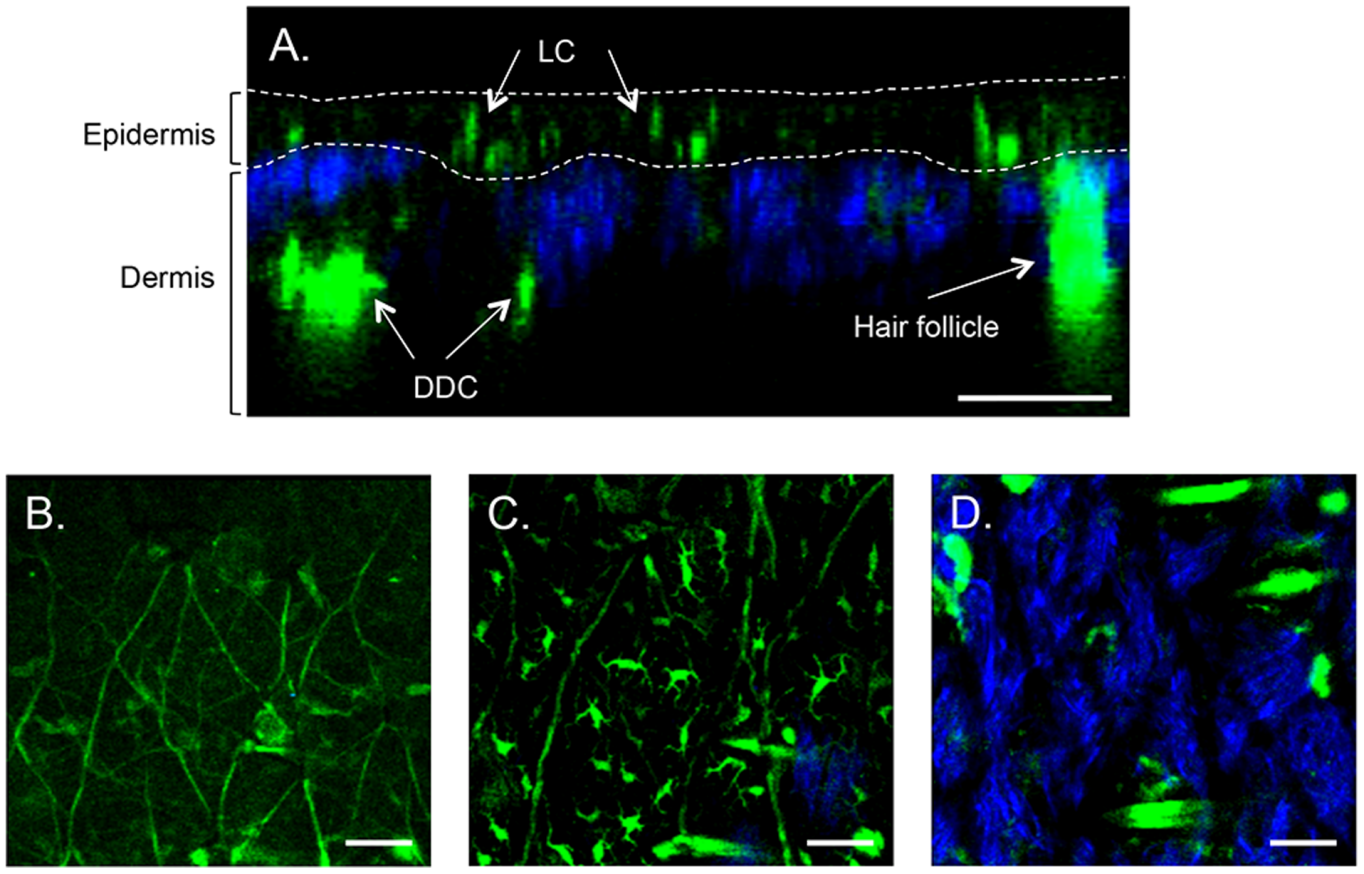
Two-photon microscopy (2 PM) visualization of Langerhans and dermal dendritic EYFP-CD11c^+^ cells in mouse skin. (A) XZ cross-sectional image of intact skin. XY images of (B) skin surface autofluorescence, (C) Langerhans cells (LC) localised in the epidermis and (D) dermal dendritic cells (DDC) in the dermal layer. Scale bar is 50 µm.

The two main populations of skin CD11c^+^ APC, the LC and DDC, reside in distinct locations (Fig. 1. and Movie S1). Two criteria were used to distinguish LC from DDC; location within the skin and physical morphology. LC were widespread in the epidermis [40] which could be clearly identified by the absence of collagen fibres (Fig. 1). Figure 1 B, C and D are XY images taken at the skin surface, through the epidermis and through the dermis, respectively. The unique morphology of LC, with their dendrites extending from the cell body, is clearly visible in Fig. 1 C. DDC had no visible dendrites and were found in the dermis (Fig. 1 D).

After MN insertion, the epidermis and the dermis were transiently disrupted (Movie S2). Due to the technical requirements for application of formulations and set-up of the microscope, the first images were taken 30–40 minutes after TCI. Formulation was observed to penetrate from the micro-channel over time and DDC were found in close proximity to areas of formulation (Movie S2 and [38]).

**Figure 2:**
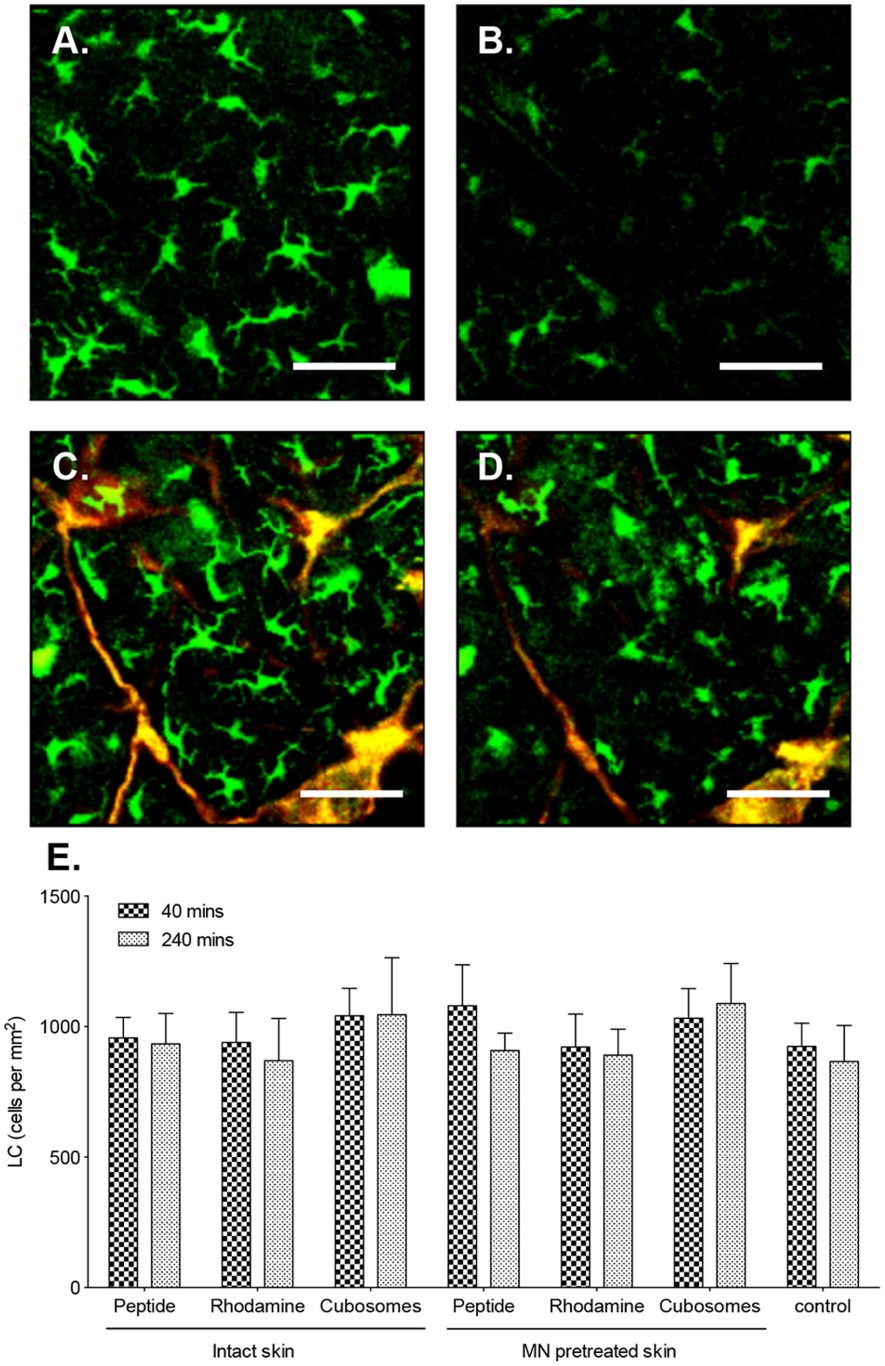
Impact of TCI on LC. Two-photon microscopy (2 PM) visualization of the distribution of LC (green) and formulation (red) in the epidermis of unimmunized mice (A, B) or following application of (C, D) TMR-SIINFEKL-loaded cubosomes for 40 min (A, C) and 240 min (B, D). Images are representative of groups of three mice. Scale bar is 100 µm. (E) The number of LC/mm^2^ in the epidermis in untreated controls (control) or following the application of aqueous TMR-SIINFEKL (peptide), rhodamine-labeled cubosomes (rhodamine) and TMR-SIINFEKL-loaded cubosomes (cubosomes) in the presence and absence of MN for 40 and 240 min. Cells were quantified from three randomised images from one mouse per group in three independent experiments. Data presented are the mean+SD.

The physical morphology and number of the LC in intact and MN pretreated skin before and after immunisation was examined (Fig. 2). The cells were non-motile (determined from time series analysis over 4 hours) and had 4–5 dendrites projecting from the body of the cell. LC density in untreated skin was determined to be approximately 980 cells/mm^2^. Upon application of the vaccine to the skin, no change in LC morphology or density could be seen (Fig. 2 D and E) over the time period investigated (40–240 minutes). By 240 min fluorescence of the LC in the skin was reduced in all samples (Fig. 2 B and D) due to photobleaching. Pretreatment of skin with MN before immunization similarly did not impact on LC density (Fig. 2 E.).

Subsequently, the ability of LC to capture formulation was examined. Detailed analysis of images using ImarisColoc (Bitplane) was carried out to quantify LC-antigen colocalization. Pearson’s coefficient was used to describe the correlation of the intensity distribution of the green fluorescence of the LC and the red fluorescence from the vaccine formulations. A Pearson’s coefficient of 1 indicates a significant positive correlation; 0 indicates no significant correlation and −1 indicates complete negative correlation. Values between 0.5 and 1 would indicate formulation-cell colocalization [41]. The calculated Pearson’s coefficient of LC from all vaccine experiments was zero throughout the experimental period. It was therefore established that no detectable formulation-LC colocalization occurred.

**Figure 3:**
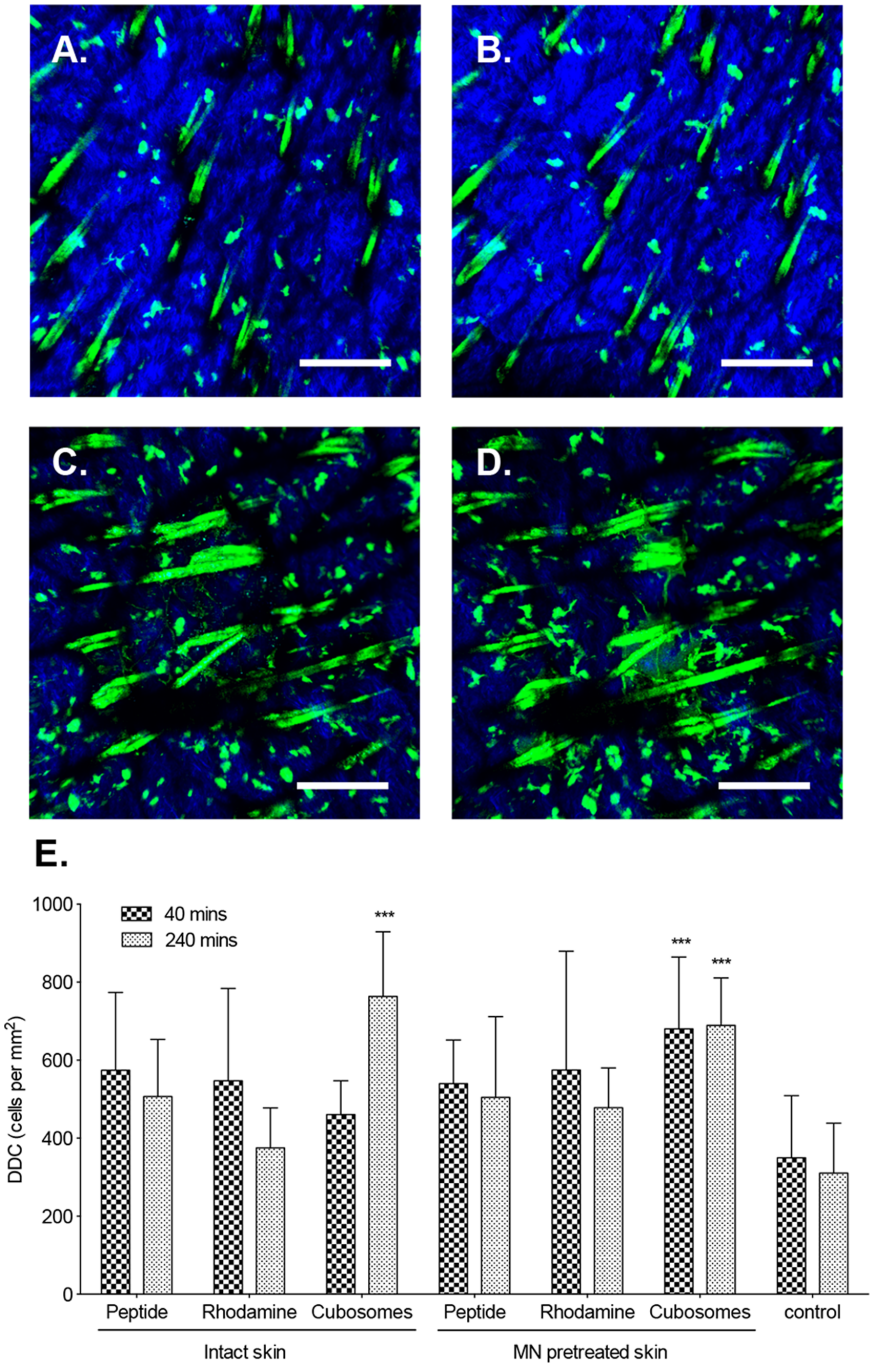
Impact of TCI on DDC. Two-photon microscopy (2 PM) visualization of DDC (green) and collagen (blue) in the dermis in unimmunized mice (A, B) or following application of MN and TMR-SIINFEKL-loaded cubosomes (C, D) for 40 min (A, C) and 240 min (B, D). Images are representative of groups of three mice. Scale bar is 200 µm. (E) The number of DDC/mm^2^ in untreated controls (control) or following the application of aqueous TMR-SIINFEKL (peptide), rhodamine-labeled cubosomes (rhodamine) and TMR-SIINFEKL-loaded cubosomes (cubosomes) in the presence and absence of MN at 40 and 240 min. Cells were quantified from three randomised images from one mouse per group in three independent experiments. Data presented are the mean+SD. ***P<0.001 compared to the control group (two-tailed unpaired Student’s t-test).

DDC were easily distinguishable in the dermis of CD11c^+^-EYFP transgenic mice. The size of DDC could not be precisely measured in these highly motile cells as they were constantly changing shape and moving both horizontally and vertically through the skin, squeezing between other cells and making contact with other DDC (Movie S3). An increase in the number of DDC was found in the skin of all mice treated with formulations when compared to unimmunized skin (control) at 40 min and 240 min following TCI (Fig. 3). However, this was significant only for mice immunized using TMR-peptide in cubosomes 240 min following immunization (P<0.001). MN pretreatment resulted in a significant increase in DDC at both 40 and 240 min post-application of TMR-peptide in cubosomes (P<0.001).

**Figure 4:**
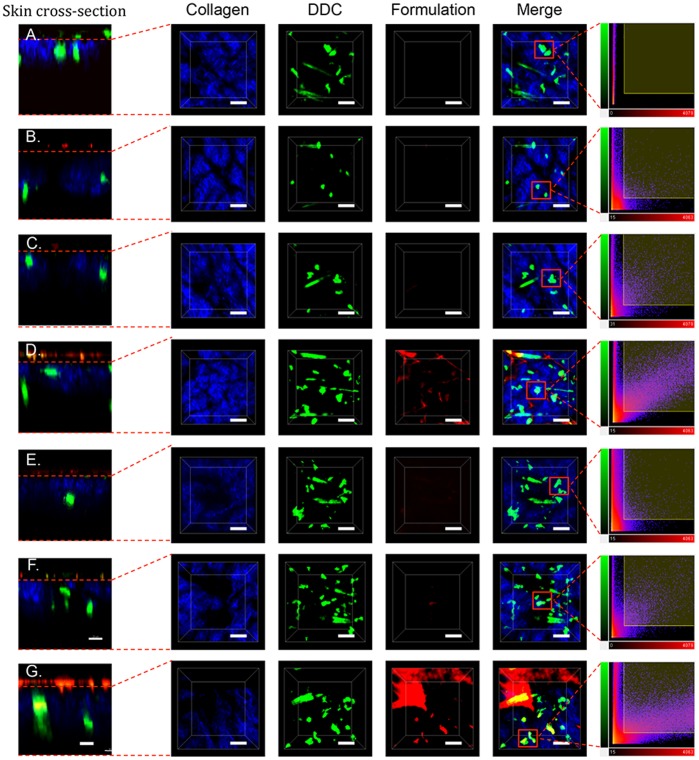
Formulation-DDC colocalization. Two-photon microscopy (2 PM) visualization of collagen (blue), DDC (green) and vaccine (red) in the dermis of untreated mice (A) or 40 min following application of an aqueous TMR-SIINFEKL mixture (B, E), rhodamine-labelled cubosomes (C, F), or TMR-SIINFEKL-loaded cubosomes (D, G) to intact (B-D) or MN pretreated (E-G) skin. Cross sections (XZ, far left) and 3-D images (XYZ) are shown as well as merged images and scatter 2D plots of voxel intensities (far right panel) in the red and green channels for selected DC. Scale bar is 100 µm.

**Figure 5:**
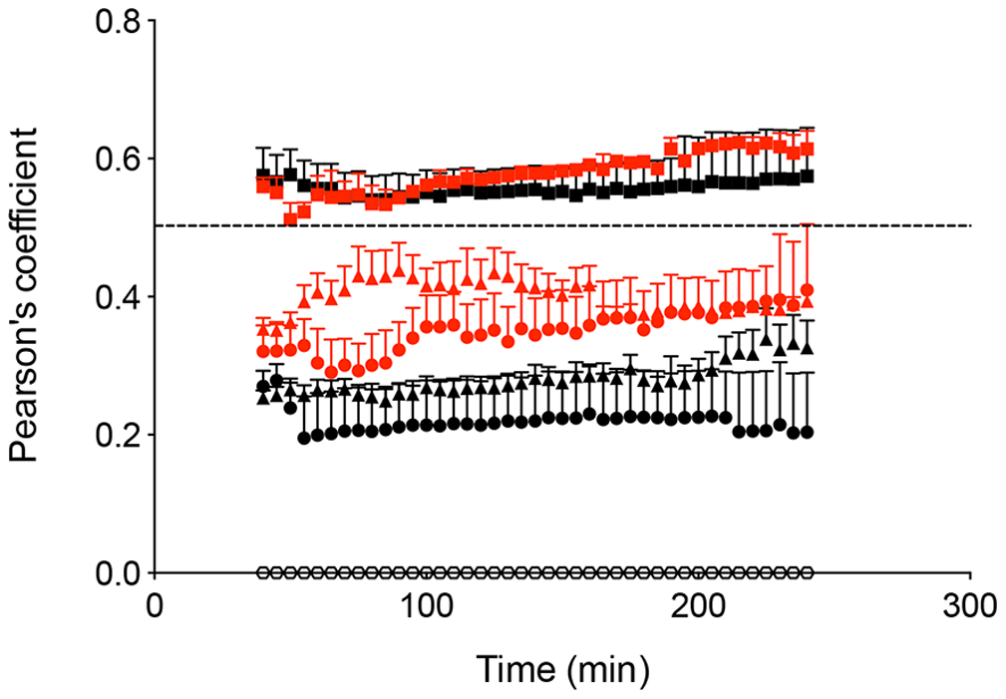
DDC-formulation colocalization. Pearson’s correlation coefficient calculated from skin treated with: an aqueous peptide mixture (•), rhodamine-labeled cubosomes (▴) and TMR peptide-loaded cubosomes (▪). The black and red colour represent intact and MN pretreated skin respectively. Unimmunized intact skin served as control (○). Cells were quantified from three randomised images from one mouse in three independent experiments. Data presented are the mean+SEM (n = 9).

Formulation-DDC colocalization was then examined by 2 PM. XYZ sections of the dermis (Fig. 4) were examined in detail 40 min after TCI. Formulation of the vaccine into cubosomes resulted in the highest levels of antigen being detected in dermis. Antigen-DDC colocalization was examined in selected cells (Fig. 4 far right column) through 2D plots of voxel intensities. Areas of high fluorescence intensity (due to autofluorescence of skin appendages) were avoided. This analysis confirmed antigen (red channel)-DDC (green channel) colocalization. Quantitative analysis of DDC-formulation colocalization was performed and expressed in terms of a Pearson’s correlation coefficient (Fig. 5) against time (40–240 min). The TMR-peptide in cubosome formulation achieved a Pearson’s coefficient above 0.5 after application to both intact and MN pretreated skin at all time points assessed.

**Figure 6:**
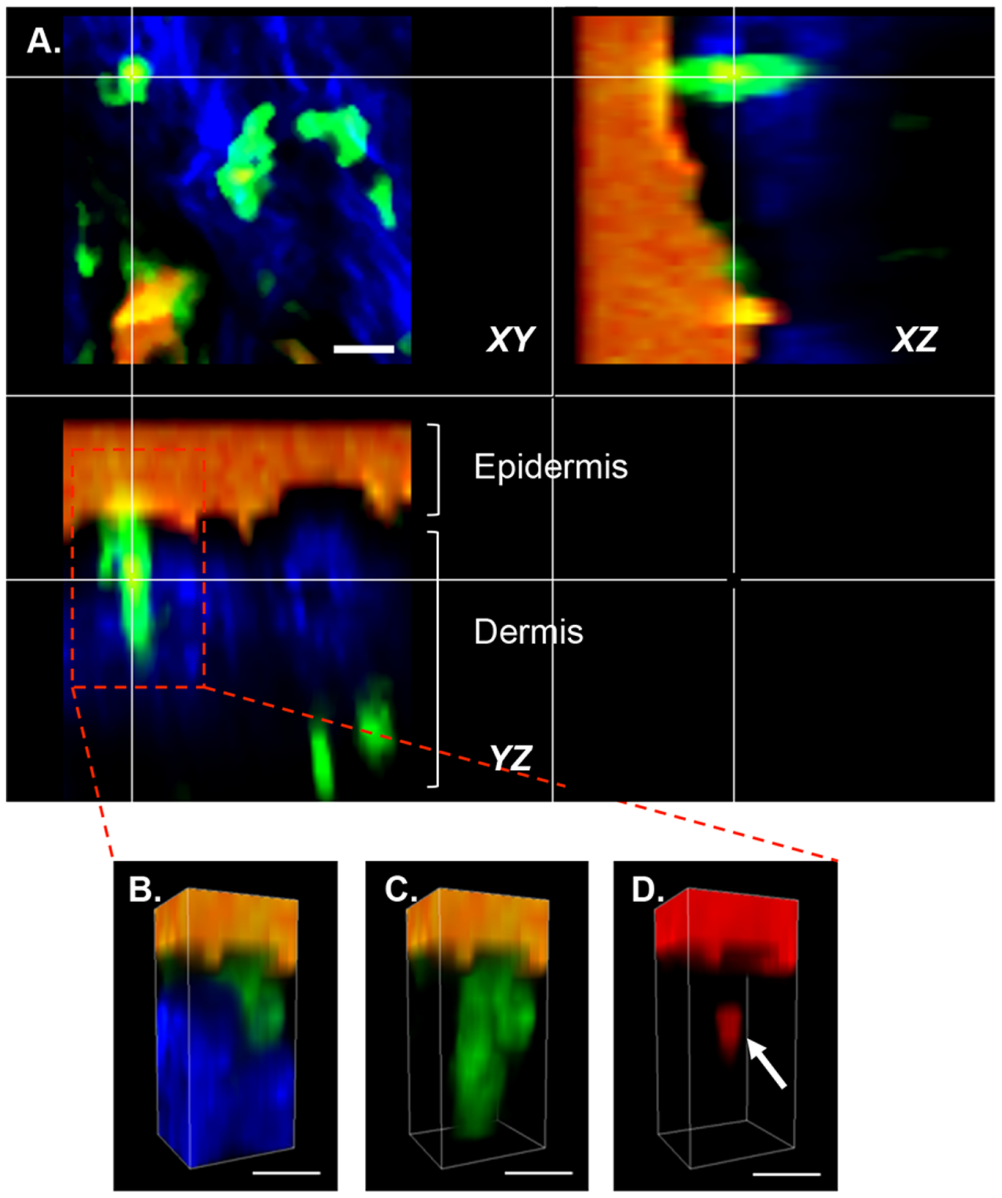
Two-photon microscopy (2 PM) visualization of DDC uptake of antigen. (A) Orthogonal views (XY, XZ, YZ) through a DDC illustrating colocalization of DDC (green), TMR-SIINFEKL (red) and collagen (blue). Colocalization is visible as a yellow colour and orange represents the leakage of intense red fluorescence into the green channel. (B–C) 3D images of the DDC from (A). All channels are shown in (B), blue has been dropped out in (C) and both blue and green in (D). TMR-SIINFEKL uptake by the DDC is shown in (D) indicated by arrow. Scale bar is 20 µm.

Interestingly, DDC could also be found extending into the epidermis in XZ cross-sectional images (Fig. 6 and Movie S4) capturing and taking up formulations from the upper layers of the skin.

### Minor Population of Langerhin^+^DDC Takes up Antigen Following TCI

**Figure 7:**
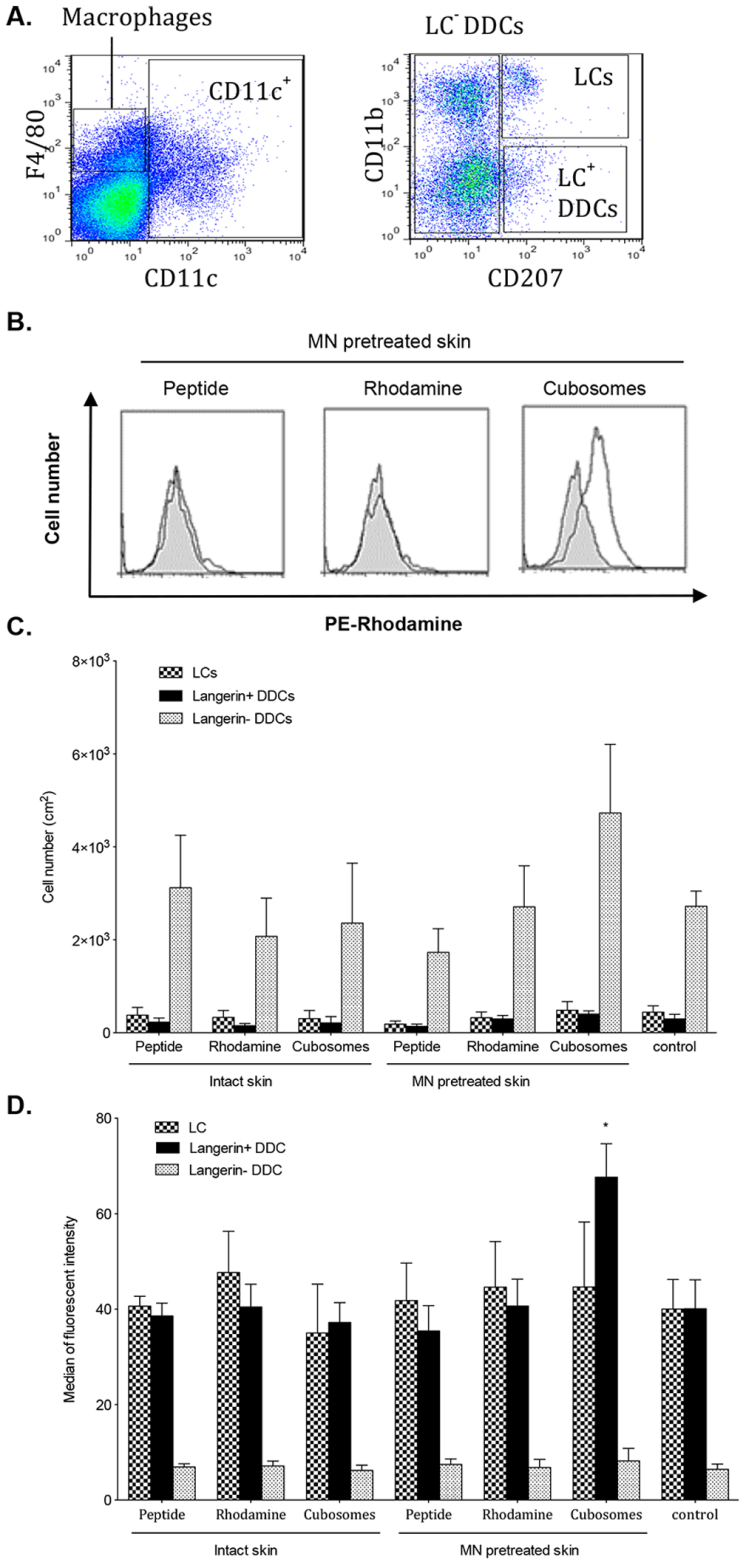
Antigen uptake by skin APC. (A) Representative dot plots showing gating for flow cytometric analysis. CD11c^hi^ DC cells were selected and further gates determined on the basis of CD207 and CD11b expression. (B) Representative histograms of the uptake of vaccine; TMR-SIINFEKL (peptide), rhodamine-labeled cubosomes (Rhodamine) or TMR peptide-loaded cubosomes (cubosomes) by Langerin^+^ DDC following TCI. The solid grey histograms are Langerin^+^ DDC from unimmunised controls and the open histograms are Langerin^+^ DDC from immunised mice. (C) Number of LC, Langerin^+^ DDC and Langerin^−^ DDC at 240 min in untreated control mice (control) or in mice immunised with aqueous TMR-SIINFEKL (peptide), rhodamine-labeled cubosomes (rhodamine) and TMR-SIINFEKL-loaded cubosomes (cubosomes) in the presence and absence of MN pretreatment. (D) Uptake of antigen (median fluorescence intensity) by LC, Langerin^+^ DDC and Langerin^−^ DDC at 240 min in untreated control mice (control) or in mice immunised with aqueous TMR-SIINFEKL (peptide), rhodamine-labeled cubosomes (rhodamine) and TMR-SIINFEKL-loaded cubosomes (cubosomes) in the presence and absence of MN pretreatment. The results of the antigen uptake are given as. Data are mean+SEM and are representative of three independent experiments with 5 mice per group. *P<0.05 (two-tailed unpaired Student’s t-test).

APC were isolated from skin immunized transcutaneously in the presence and absence of MN pretreatment or from unimmunized control mice. Skin DC were identified based on CD11c expression and were then gated based on CD207 and CD11b expression (Fig. 7 A) into LC (CD207^hi^, CD11b^hi^), langerin^+^ DDC (CD207^hi^, CD11b^lo^ [1]) and langerin^−^ DDC (CD11b^ hi/lo^ CD207^ lo^ [7], [42]). LC comprised approximately 8–10% of the isolated skin DC (Fig. 7 C). A small population of langerin^+^ DC was also found in the full-thickness skin (3–4% of total DC in the skin) in agreement with previously published studies [43]. The largest population of DC in the skin were the Langerin^−^ DDC population (∼70%). Following formulation application, while there was no significant change in the number of any of the DC subsets (Fig. 7 C), uptake of fluorescent peptide by skin DC (Fig. 7 B and D) was detected in the minor langerin^+^ DDC subset in mice treated with MN and peptide-loaded cubosomes (P<0.05). Consistent with the 2 PM antigen-DC colocalization studies, no uptake by LC was detected.

**Figure 8:**
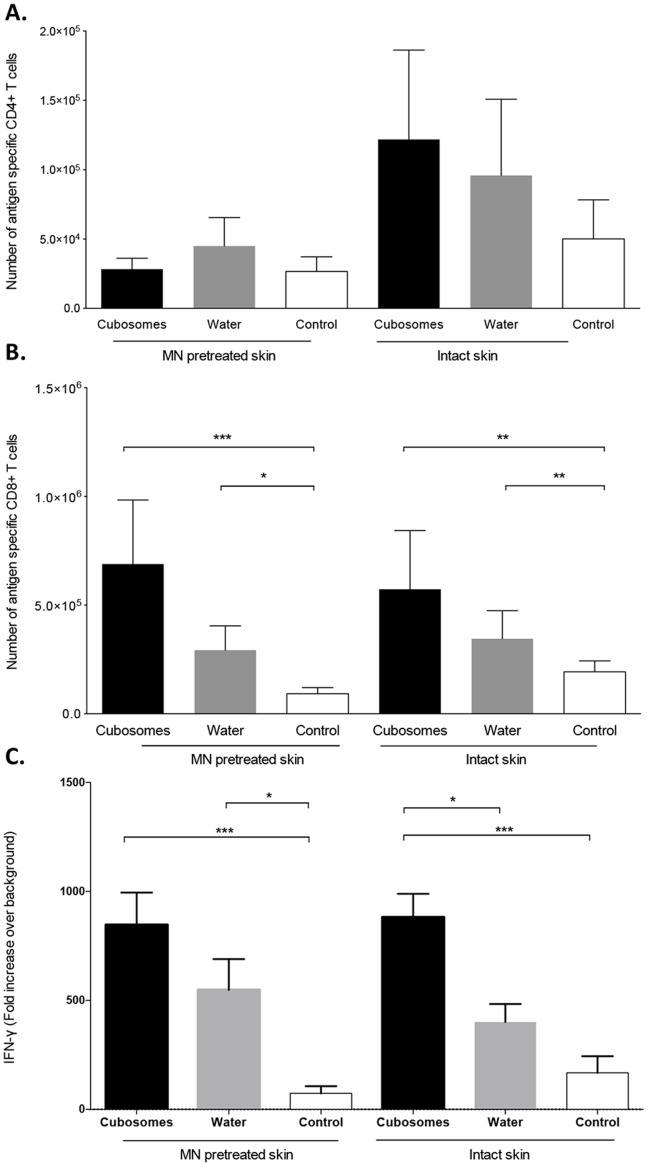
Immune responses stimulated by TCI. Number of transgenic Vα2^+^Vβ5^+^ CD4^+^ (A) and Vα2^+^Vβ5^+^ CD8^+^ (B) T cells in lymph nodes of mice vaccinated with 100 µg of CD4^+^ peptide, 100 µg of CD8^+^ peptide, 40 µg of QA and 20 µg of MPL formulated in cubosomes (Cubosomes) or in Milli-Q water (Water) or with cubosomes containing only MPL and QA (Control) in the presence and absence of MN pretreatment. (C) Production of interferon-γ (IFN-γ), expressed as fold increase over background, by cells isolated from lymph nodes of mice and re-stimulated *in vitro* with OVA or media (as a negative control). Data shown are the mean+SEM from three independent experiments with 3–4 mice in each experiment. *P<0.05, **P<0.01 and ***P<0.001 (two-tailed unpaired Student’s t-test).

The ability of TCI with the OVA CD4 and CD8 minimal peptide epitopes either delivered in water or in cubosomes, with or without MN pretreatment to induce immune responses following a prime-boost regime was assessed (Fig. 8). The number of antigen-specific CD4^+^ T cell in the lymph nodes (Fig. 8 A) was low and not above that found in control mice immunised with a control cubosome formulation containing the adjuvants but no antigen (P>0.05). Mice immunized transcutaneously with peptide in water or peptide formulated in cubosomes had a measureable antigen-specific CD8^+^ T cell expansion as compared to the control cubosome formulation (Fig. 8 B). The combination of pretreatment with MN before the application of the formulation did not significantly enhance the antigen specific expansion of CD8^+^ T cells as compared to formulation applied to intact skin. Similar results were found in a functional assay where the ability of lymph node cells to produce IFN-γ upon restimulation with antigen was examined. Lymphocytes from mice immunized using MN and either cubosome or peptide in water produced significant levels of antigen specific IFN-γ (Fig. 8 C). However when the formulations were applied to intact skin only lymphocytes from mice immunised with cubosomes produced significant levels of IFN-γ (P<0.05).

## Discussion

The aim of this study was to investigate the interface between TCI and the immune system using 2 PM and flow cytometry. TCI was accomplished using a lipid nanocarrier in combination with MN. Cubosomes were chosen as the carrier due to their ability to promote penetration of vaccine into the skin *in vitro* [11]. MN were used to temporarily disrupt the skin barrier and improve vaccine delivery to the epidermis and the dermis where LC and DDC reside. The synergistic effect of combining lipid particles and MN on immunological responses has been previously reported by Hirschberg *et al.* [44]. They indicated that the induction of antibody responses occurred only when mice were immunized with a combined approach using both vesicles and MN. Zaric *et al*. [45] also reported that robust immune responses were generated by utilizing MN to deliver vaccine-loaded poly-D-L-lactide-co-glycolide (PGLA) nanoparticles to the skin APC.

LC present in the epidermis are the first APC that transcutaneously applied vaccine antigens should encounter. However in the early stages of immunization examined here, LC were not found to be motile, the number of LC was not altered in response to the application of formulation and no antigen-LC colocalization could be detected either by 2 PM or flow cytometry. Sen *et al.* [46] similarly found that LC were completely immotile until 48 hr after the injection of adjuvants such as CpG and LPS and even then only a subpopulation of LC (approximately 15%) moved at a very low velocity. Studies of TCI using MN in human skin explants have also shown that LC responses occur over 24 to 48 hours [18], [47]. The role of LC in the initiation of immune responses is conflicting and incompletely understood. While LC have been shown to be able to extend their dendrites into the SC layer to capture antigen [48] and to be able to efficiently take up soluble antigen [49] other studies have shown they are not involved in priming immune responses in skin but are perhaps involved in tolerance [2]. The fact that LC do not appear to be involved in the immediate response in interesting and is perhaps suggestive of a more regulatory role.

TCI using peptide loaded-cubosome formulations was however found to have an impact on DDC recruitment and this occurred earlier if MN pretreatment was employed. Two adjuvants with different immune activatory activities were included in the formulation in order to optimize immune stimulation. QA is a hydrophilic TLR-independent adjuvant that has recently been reported to activate the NLRP3-inflammasome [50] while MPL is a lipophilic TLR-4 ligand [51], [52], [53]. Cubosomes were chosen as the delivery system as their biphasic structure facilitates entrappment of both adjuvants and the antigen. In addition particulate delivery systems have been shown themselves to activate the inflammasome [54]. Activation of the NALP3-inflammasome leads to release of IL-1β, a potent proinflammatory cytokine, which results in the recruitment of immature DC [55], [56]. TLR-4 agonists activate NF-κB, a pro-inflammatory gene transcription regulator, which results in the up-regulation of the co-stimulatory molecules CD80 and CD86 [57] and the production of IP-10/CXCL10 chemokines responsible for Th1 recruitment [58]. In this study we observed DC recruitment occurring 40 min after immunization with TMR-peptide in cubosomes (when MN were utilized) and within 4 hr when the vaccine was applied to the intact skin. This only occurred if the vaccine antigen and adjuvant were loaded into cubosomes, not when they were applied as an aqueous mixture. Previous investigations into the mechanism of vaccine permeation into skin revealed that the lipophilic cubosomes are retained in the microchannels formed by MN creating an immunestimulatory depot and also facilitating the movement of unentrapped peptide antigen and adjuvant into the deeper layers of the skin to interact with APC [38]. The kinetics of the response is similar to that found following topical application of measles virus nucleoprotein onto mucosal tissue where DC were found to be recruited rapidly, peaking at 2 hr, while DC migration to the draining lymph node took place over 24 hr [59]. Interestingly, rhodamine labelled cubosomes containing the adjuvants MPL and QA but no antigen did not significantly effect DDC recruitment. This requirement for antigen suggests that DC-T cell interactions are required for the observed DDC recruitment. The skin contains large numbers of skin resident memory T cells [60] and naïve T cells [61]. It would be of interest to examine the interactions occurring between DC and T cell subsets in the skin using 2 PM.

Antigen-DDC colocalization could be visualized by 2 PM and confirmed by calculation of Pearson’s correlation coefficient, in skin treated with TMR-peptide in cubosomes in the presence and absence of MN pretreatment. Current dogma suggests that DDC are unable to penetrate into the epidermis because of the barrier provided by the epidermis-dermis tight junction [62], although it has been reported that DC cluster around hair follicles and that dendrites can penetrate the basement membrane to sample antigen present in the epidermis [63]. Flow cytometry suggested that it was the langerin^+^ DDC subset that was taking up antigen. As the vaccine was not actively targeting these cells the preferential uptake of the vaccine must be related to the phenotype or physical location of this population of cells. Bursch *et al* [63] reported that langerin^+^ DDC have been identified penetrating into the epidermis therefore it may be that the physical location of these cells results in the preferential uptake. This population of cells has additionally been reported to trigger Th1 immune responses and induce predominantly cellular immunity, including antigen-specific CD8^+^ T cell [6]. It is possible that by utilizing MN the vaccine is targeted to a different subset of APC than it would be if applied to intact skin and that a qualitatively different immune response is produced.

Immune responses to vaccines applied transcutaneously to mice were examined and while there were perhaps some minor differences in the responses induced by the vaccines, all vaccines were able to stimulate weak but detectable responses. This is a limitation of using mice to examine immune responses to vaccines applied transcutaneously, as vaccination is much more effective due to the thin SC (approximately 9 µm in mice and human 20 µm in humans [64]) and high number of hair follicles (approximately 660/cm^2^ in mice and 11/cm^2^ in humans [65]). An additional issue is the removal of hair by methods such as plucking, shaving or through the use of a depillatory cream. All these methods, including the depillatory cream used here, can act to enhance vaccine penetration [66], [67]. Therefore while mice are an invaluable tool for investigating cellular interactions occurring in the skin they are less useful for evaluating the immunological activity of potential vaccine formulations.

In conclusion the use of a lipid based delivery system in combination with MN can potentiate immune responses to peptide antigens delivered transcutaneously by facilitating uptake of vaccine by dermal dendritic cells. Further preclinical studies in more appropriate animal models (for example miniature pigs) or clinical studies in man will be necessary in order to determine the full potential of such vaccine formulations.

## Supporting Information

Movie S1 **Two-photon microscopy (2 PM) visualization of EYFP-CD11c^+^ in mouse skin.** Collagen is shown as blue fluorescence and green fluorescence represents stratum corneum or hair follicle autofluorescence and EYFP-CD11c^+^ cells.
(MP4)

Movie S2 **Transient formation of a microchannel and penetration of vaccine following application of peptide loaded cubosomes (shown in red) to MN pretreated skin.** A 3D image (top) and XZ cross sections (bottom) are shown. Collagen is visible as blue fluorescence and green fluorescence represents EYFP-CD11c^+^ cells. Bright yellow florescence is a result of autofluorescence of hair follicles. EYFP-CD11c^+^ DDC can be seen in close proximity to the vaccine formulation.
(MP4)

Movie S3 **Rapid movement of DDC in normal skin.** Collagen is shown as blue fluorescence and green fluorescence represents EYFP-CD11c^+^ cells.
(MP4)

Movie S4 **Uptake of vaccine by DDC.** A highly motile DDC can be seen taking up vaccine from the epidermis. Peptide is visible as red fluorescence, collagen is shown as blue fluorescence and green fluorescence represents EYFP-CD11c^+^ cells. Areas of antigen/DDC colocalization are yellow.
(MP4)
